# Targeted Therapies for Rheumatoid Arthritis in Super‐Elderly Society: Insights From FIRST Registry, Japan

**DOI:** 10.1111/1756-185X.70232

**Published:** 2025-04-24

**Authors:** Koshiro Sonomoto, Yoshiya Tanaka

**Affiliations:** ^1^ Department of Clinical Nursing, School of Health Sciences University of Occupational and Environmental Health, Japan Kitakyushu Japan; ^2^ The First Department of Internal Medicine, School of Medicine, University of Occupational and Environmental Health, Japan Kitakyushu Japan; ^3^ Department of Molecular Targeted Therapeutics University of Occupational and Environmental Health, Japan Kitakyushu Japan

**Keywords:** aging population, infection risk, malignancy management, rheumatoid arthritis, targeted therapies, treatment optimization

## Abstract

As the world's most rapidly aging society, Japan has faced significant challenges in the management of rheumatoid arthritis (RA) in elderly patients. Biologic and targeted synthetic disease‐modifying antirheumatic drugs (b/tsDMARDs) have improved disease control, but concerns remain regarding the risk of infection and malignancy, especially in the elderly population. This review presents real‐world data from FIRST registry, a prospective cohort following a total of 5500 patients over 20 years, and highlights strategies to balance efficacy and safety. Registry shows an increasing age of patients initiating b/tsDMARDs and an increase in comorbidities such as cardiovascular disease, chronic kidney disease, and malignancies. Infection control—including prophylaxis against pneumocystis pneumonia, screening for non‐tuberculous mycobacteria, and vaccination—has been associated with a lower incidence of serious infections. Similarly, a proactive malignancy management strategy that includes computed tomography (CT) screening and multidisciplinary care has been correlated with improved detection of early‐stage cancers and fewer treatment discontinuations due to malignancy. While these findings represent associations rather than direct causal effects, they reflect the evolving landscape of RA management over two decades. Meanwhile, the registry highlights unmet needs, particularly in patients with prior b/tsDMARD exposure, who often experience lower response rates. In addition, while clinical remission rates have improved, functional outcomes remain suboptimal, particularly in older patients. To address these challenges, personalized treatment selection is being explored. By sharing Japan's experience in managing RA in an aging society, this review provides insights into strategies that may be applicable to other aging populations worldwide.

## 1 Introduction

As Japan leads the world in population aging, the healthcare landscape is facing unprecedented challenges, particularly in the management of chronic diseases such as rheumatoid arthritis (RA). In Japan, demographic change has led to a marked increase in the proportion of RA patients aged 65 years and older, many of whom are further burdened by comorbidities such as chronic kidney disease, cardiovascular disease, lung disease, and a history of serious infections [1]. These conditions not only complicate the management of RA, but also increase the risks associated with intensive RA treatments, making it particularly challenging to achieve optimal outcomes in an aging population [2].

Molecular targeted therapies, including biologic and targeted synthetic disease‐modifying antirheumatic drugs (b/tsDMARDs), have transformed the treatment of RA by offering the potential for disease control and remission. However, the immunosuppressive effects of these therapies raise significant safety concerns, particularly in older patients with compromised health. For example, the use of b/tsDMARDs has been associated with increased susceptibility to infection [3], while the use of Janus kinase inhibitors (JAKi) has been suggested to increase the risk of malignancy [4]. Thus, the management of RA in an aging population requires a delicate balance: treatments must be effective enough to control disease activity and prevent joint damage, yet cautious enough to minimize adverse events and protect the overall well‐being of the patient.

In the face of these challenges, our approach to RA management emphasizes a comprehensive strategy that carefully balances safety and efficacy [1]. This includes tailored use of molecular targeted therapies, proactive infection prevention, and close monitoring for malignancy. At the same time, b/tsDMARDs are administered promptly to patients without uncontrollable risk factors to ensure timely disease control and prevent undertreatment. By integrating these strategies into daily practice, we aim to maximize both the efficacy and safety of RA treatment in elderly patients, addressing unmet medical needs and working toward sustained functional improvement [1].

This review summarizes findings from real‐world data within the oldest population, with a focus on infection and malignancy risk management, lessons learned from bone density outcomes, and the importance of addressing unmet needs. By highlighting Japan's experience in managing RA in an aging society, this review aims to provide valuable insights for countries worldwide facing similar demographic trends and healthcare challenges.

## 2 The Advancement of FIRST Registry With a Growing Population

**FIGURE 1:**
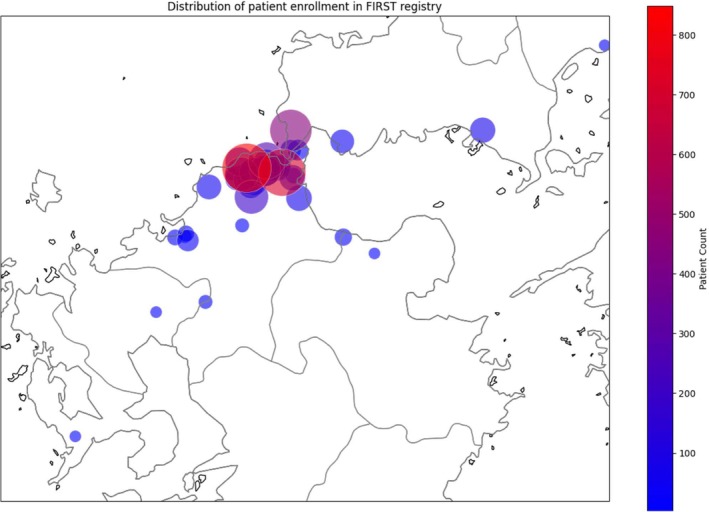
Geographical distribution of patients in FIRST registry. Maps show the location and size of the FIRST registry population.

FIRST registry is a real‐world prospective cohort established at our institute and 28 surrounding institutions in nine municipalities in six prefectures (Figure 1) [1]. It was initiated in 2003 with the introduction of infliximab to determine the optimal use of bDMARDs in the treatment of RA. Over the past 20 years, the registry has followed approximately 5500 patients, providing valuable insights into the evolving demographics and treatment outcomes of RA patients receiving b/tsDMARDs.

Data from FIRST registry show a steady increase in the age of RA patients starting b/tsDMARDs, with a notable increase in patients aged 65 years and older. The median age increased from 51.9 years in 2003 to 64.3 years in 2023, and the proportion of patients aged 65 years and older increased from 7% to 54% [1]. This aging population presents unique challenges, as older patients are more likely to have multiple comorbidities. Over the same period, the prevalence of cardiovascular disease increased from 2% to 9%, chronic kidney disease from 2% to 25%, respiratory disease from 11% to 36%, and a history of malignancy from 2% to 13% [1]. These comorbidities complicate the management of RA by limiting treatment options and increasing the risk of adverse events.

Despite these challenges, data from FIRST registry show remarkable improvements in outcomes and reductions in adverse events over time [1]. Over the past 20 years, the percentage of patients achieving clinical remission 6 months after starting b/tsDMARDs has increased from 10.0% in the era when only tumor necrosis factor inhibitors (TNFi) were available (2003–2007) to 26.4% in the era when multiple treatment options—including TNFi, IL‐6 receptor inhibitors (IL‐6Ri), CTLA4‐Ig, and JAKi—became available (2013 onwards). In addition, adverse events requiring b/tsDMARD discontinuation decreased from 5.6 per 100 person‐years (py) to 3.7 per 100 py, with reductions in both infectious and non‐infectious adverse events [1].

This shift toward safer, more effective treatment outcomes raises questions about the factors driving these improvements. In the next section, we will review our strategies for reducing the risk of infection and managing malignancy, both of which play a critical role in improving the safety and efficacy of RA treatment in an aging population.

## 3 Challenge Against Risk—Infection

### 3.1 Prophylaxis Against Pneumocystis Pneumonia

One major infection of concern in RA management is pneumocystis pneumonia (PCP), an opportunistic infection with high mortality, particularly in patients with autoimmune rheumatic diseases. Despite this risk, international guidelines offer limited recommendations for PCP prophylaxis, focusing primarily on patients receiving high‐dose glucocorticoids—a strategy that is less applicable to RA, where such treatment is rarely used. Recognizing this gap, we developed a tailored prophylaxis strategy for RA patients undergoing b/tsDMARD therapy [5].

Using data from FIRST registry, we first established criteria to guide PCP prophylaxis. The first version of these criteria (version 1) focused on key risk factors such as age, presence of lung disease, and any glucocorticoid use [5]. This strategy was highly effective, reducing the incidence of PCP from 1.6% to 0.4% and the mortality rate from 7.8 to 1.7 per 10 000 patients with RA. However, evolving patient demographics and new evidence led us to refine the criteria: the updated criteria (version 2) now include a broader range of PCP risk factors, including age, serum albumin levels, immunoglobulin G (IgG) levels, body mass index (BMI), lung disease, lymphocyte count, and glucocorticoid use [5].

Our data also show that sulfamethoxazole‐trimethoprim (SMX‐TMP) prophylaxis is more effective than pentamidine inhalation, establishing SMX‐TMP as the preferred prophylactic agent [5]. Although SMX‐TMP can occasionally lead to treatment discontinuation due to adverse events, registry data show a high retention rate (unpublished data). The regular use of a reduced SMX‐TMP regimen likely contributes to this high adherence [5]. As a result, the widespread adoption of this SMX‐TMP‐based strategy (applied to approximately half of patients receiving b/tsDMARDs) has correlated with a sustained reduction in PCP incidence, underscoring the impact of this targeted approach on patient safety.

### 3.2 Early Detection and Management of Non‐Tuberculous Mycobacterial Infections

In addition to PCP, non‐tuberculous mycobacterial (NTM) infections pose an additional challenge for RA patients undergoing immunosuppressive treatment. The prevalence of NTM infections in RA patients is notably higher than in the general population, with a significantly increased risk of mortality once the infection becomes symptomatic. Results from FIRST registry showed that screening RA patients with chest computed tomography (CT) scans prior to initiating b/tsDMARD therapy was highly effective in detecting NTM infections [6]. Of the 4447 patients who received a CT scan prior to treatment intensification with b/tsDMARDs, 33 were diagnosed with NTM, most of whom were asymptomatic (84.8%), with 42.4% having lesions that were not detectable by chest X‐ray. These undetected lesions could otherwise progress unnoticed, potentially leading to a poor prognosis.

Early detection allowed for timely treatment adjustments, allowing patients to safely continue their RA therapy without worsening NTM disease activity. A 24‐month follow‐up showed similar levels of disease activity in patients with and without NTM, underscoring the importance of early detection in maintaining stable RA treatment [6].

### 3.3 Vaccinations

Vaccinations play a critical role in managing the risk of infection in RA patients, especially those with advanced age. Our RA management strategy emphasizes both influenza and pneumococcal vaccinations, which are essential to prevent serious respiratory infections that could lead to delays in RA treatment [1]. Given the immunocompromised state induced by RA treatments, annual influenza vaccination is recommended to strengthen immunity during peak infection seasons. Pneumococcal vaccination is also emphasized, especially for older adults who are at higher risk for invasive pneumococcal disease. Real‐world data from FIRST registry further support the benefits of these vaccinations, showing reduced rates of serious infections (2.1/100 py in 2003–2007 to 0.7/100 py in 2013–2023) among patients receiving b/tsDMARDs as vaccination coverage has increased [1].

### 3.4 Minimizing Glucocorticoids Use

In addition, minimizing the use of oral glucocorticoids may reflect our broader commitment to reducing the risk of infection in the treatment of RA. While glucocorticoids are commonly used in RA for their anti‐inflammatory effects, they are also associated with increased susceptibility to infection due to their immunosuppressive properties [7]. According to FIRST registry, reduced glucocorticoid dependency (with 67% of patients on concomitant glucocorticoids in 2003 compared to 21% in 2023) has been associated with a reduction in the rate of serious infections [1, 8]. By carefully managing glucocorticoid use, our RA care model aims to balance effective disease control with minimized risk of infection.

Together, these strategies form a comprehensive infection management approach within the framework of RA treatment. By integrating prophylaxis, screening, vaccination, and minimized glucocorticoid use, RA management aims to optimize patient safety. This approach has been shown to be effective in reducing treatment discontinuations due to adverse events [1], highlighting the importance of infection control in a population where age‐related vulnerabilities and comorbidities are prevalent. As RA management continues to evolve, infection control will remain a cornerstone of safe and effective RA care in aging populations worldwide.

## 4 Challenge Against Risk—Malignancy

The chronic inflammation inherent in RA, combined with the immunosuppressive effects of RA treatments, contributes to an increased incidence of certain malignancies, including lymphoma, lung cancer, and skin cancer [4]. This malignancy risk is compounded in Japan's aging RA population, where age‐related vulnerabilities add to the complexity of balancing disease control and safety. In response, FIRST registry has pioneered a proactive malignancy management strategy that prioritizes early detection and inter‐specialty collaboration [9]. This comprehensive approach has been instrumental in improving outcomes and reducing treatment‐related risks.

### 4.1 Malignancy Risks in RA Patients

Research has shown that patients with RA have higher rates of certain malignancies compared to the general population, with significantly elevated standardized incidence ratios (SIRs) reported for malignancies such as lymphoma, lung cancer, and skin cancer [4]. These increased risks are often attributed to the pro‐inflammatory environment of RA, which may contribute to oncogenesis, as well as to the immunosuppressive effects of long‐term RA treatment. For example, while JAKis are effective in controlling RA, concerns have been raised about their association with increased cancer risk in patients with predisposing factors [4]. This risk of malignancy is of particular concern in the aging population, where weakened cellular repair mechanisms may exacerbate susceptibility, underscoring the need for robust screening and intervention strategies.

### 4.2 CT Screening for Early Detection of Malignancy

To address the increased risk of cancer in RA patients receiving b/tsDMARD therapy, a core feature of the malignancy management strategy in FIRST registry is the use of CT scans for comprehensive cancer screening [9]. Unlike standard screening practices, which often rely on physical exams and X‐rays, CT screening provides a more sensitive modality capable of detecting malignancies at earlier stages. This approach has been particularly effective in detecting lung cancer and other malignancies that may otherwise remain asymptomatic until they reach advanced stages.

In a study of 2192 RA patients who underwent CT scans before starting b/tsDMARD therapy, 1.5% of patients were diagnosed with malignancies, with a remarkable 80% of these cases detected at an early, treatable stage [9]. The sensitivity of CT screening allowed detection of malignancies missed by standard examinations. This early detection allowed patients to undergo timely surgical or pharmacologic intervention, with 82% of patients surviving for up to 10 years. In contrast, only an estimated 14% of patients with cancers detected by regular screening who did not receive early intervention survived. This finding underscores the effectiveness of CT screening in identifying occult malignancies in high‐risk RA patients, giving them a greater chance of successful treatment and improved survival.

### 4.3 The Role of Collaborative Care in Malignancy Management

Another key element of the malignancy management strategy in FIRST registry is a multidisciplinary approach that promotes close collaboration between rheumatologists, oncologists, and other specialists [9]. This team‐based care model is essential for managing the dual challenges of RA and cancer, as each disease requires specific therapeutic strategies that may affect the other. In most cases, when RA patients are diagnosed with cancer, b/tsDMARD therapy is temporarily discontinued to avoid interfering with cancer treatment. This can lead to flare‐ups of RA symptoms, so it is important for rheumatologists to manage RA carefully during this time.

Throughout cancer treatment, rheumatologists work closely with oncologists to adjust RA therapy to maintain disease control without interfering with the effectiveness of cancer treatments. Conventional DMARDs or low‐dose glucocorticoids may be used temporarily to control RA activity until cancer treatment is completed. Once the cancer is in remission, b/tsDMARDs can be reintroduced through a shared decision‐making process involving the patient, oncologist, and rheumatologist [10]. This collaborative approach ensures that RA is effectively managed without undue delays or undertreatment, preserving patients' quality of life and preventing disease progression.

While FIRST registry has documented treatment patterns in RA patients with malignancy [9], more research is needed to determine the direct impact of a multidisciplinary approach on clinical outcomes. However, this strategy is consistent with general recommendations that emphasize the importance of coordination between rheumatologists and oncologists to minimize treatment interruptions and balance RA management with cancer care [10].

### 4.4 Long‐Term Surveillance and Monitoring

In addition to initial CT screening, ongoing surveillance remains a critical aspect of malignancy management in RA patients. Rather than using CT scans for regular monitoring due to potential toxicity, the approach emphasizes a structured cancer screening program in accordance with national guidelines. This program includes regular chest X‐rays, fecal occult blood testing for colorectal cancer, and screening for stomach, breast, and cervical cancer. These standardized screenings are critical for the early detection of malignancies in RA patients, allowing for timely intervention without the added radiation exposure of frequent CT scans.

Patient education is also a key component of this long‐term surveillance strategy. Ensuring that patients understand the importance of regular screening and its role in early cancer detection empowers them to take an active role in their health. This proactive engagement contributes to consistent screening compliance, which increases the overall effectiveness of malignancy management.

Interestingly, patients who underwent initial CT screening had a 5‐year SIR of 0.35 for malignancies, which is significantly lower than that observed in RA populations who did not undergo CT screening [9]. This finding highlights the effectiveness of well‐planned initial screening combined with consistent, patient‐centered surveillance in reducing the risk of malignancy in high‐risk RA patients.

### 4.5 Impact of the Malignancy Management Strategy on RA Treatment Retention

By integrating early malignancy detection and collaborative care, FIRST registry has observed reduced discontinuation of b/tsDMARD due to adverse events, the majority of which are malignancies [1]. This strategy may also help reduce psychological distress. With routine screening and a dedicated care team overseeing both RA management and malignancy prevention, patients can approach their treatment with greater confidence, reducing concerns about undetected cancers. This reassurance promotes better adherence to RA therapy and supports overall treatment success, underscoring the value of a comprehensive and patient‐centered approach to malignancy management in RA.

### 4.6 Implications and Future Directions

The malignancy management framework established in FIRST registry provides a valuable model for other aging societies worldwide. As the RA population continues to age, managing the risk of malignancy remains a critical priority. The strategies implemented in FIRST registry—including initial CT screening, multidisciplinary collaborative care, and structured cancer screening programs—represent best practices that can be adapted to meet the needs of RA patients in different healthcare settings.

Future research may focus on refining malignancy screening criteria to optimize early detection and improve cost‐effectiveness. Such efforts could further support the effective management of RA patients while minimizing unnecessary exposure to screening‐related risks. Advances in imaging technology and the development of biomarkers may also improve early detection and allow for a more accurate assessment of malignancy risk in RA patients.

## 5 Other Comorbidities to Consider in Aging Societies

In addition to the challenges discussed above, two other comorbidities have received special attention in the FIRST registry framework: cardiovascular disease (CVD) and osteoporosis, both of which have a marked impact on patient prognosis.

### 5.1 Cardiovascular Diseases

Patients with RA have a higher risk of cardiovascular events compared to the general population [11, 12]. In East Asia, including Japan, the burden of cerebrovascular disease exceeds that of CVD, in contrast to Western countries [13]. However, as hypertension, diabetes, and hyperlipidemia become more prevalent with age, concerns about cardiovascular risk in RA patients are becoming increasingly important.

The FIRST registry framework includes a comprehensive pre‐treatment evaluation to assess both RA‐related and non‐RA‐related comorbidities to ensure that potential risks are identified prior to the initiation of b/tsDMARDs. This comprehensive evaluation includes cardiovascular risk factor assessment and screening for metabolic and other relevant conditions. Identified comorbidities are either managed directly by the treating physician or referred to a specialist as necessary. Because JAKi have a different mechanism of action than conventional bDMARDs, their safety profile has been carefully evaluated in FIRST registry, with a particular focus on the risks of infection and malignancy. As a result, JAKi have been prescribed primarily to younger patients with fewer comorbidities [1].

Oral Surveillance study [14] evaluated the safety of tofacitinib versus TNFi in RA patients aged 50 years and older with at least one cardiovascular risk factor (e.g., smoking, hypertension, dyslipidemia, diabetes, family history of premature coronary artery disease, extra‐articular RA, or history of coronary artery disease). The study failed to demonstrate non‐inferiority of tofacitinib for major adverse cardiovascular events (MACE), and there was a more prevalent incidence of thrombosis in the tofacitinib group. These findings raised concerns about the cardiovascular and thrombotic risks associated with JAKi [15].

Leaving RA patients with these comorbidities untreated or with residual disease activity may further increase their cardiovascular risk. Therefore, treatment should be optimized based on a treat‐to‐target (T2T) strategy, with careful selection of b/tsDMARDs in patients at high vascular risk. In response to the Oral Surveillance findings, FIRST registry has included MACE and thrombotic risk assessment in its JAKi eligibility criteria. To date, the registry has not observed a remarkable increase in MACE or thrombosis associated with JAKi use.

### 5.2 Osteoporosis and b/tsDMARDs

Osteoporosis is commonly associated with RA and has become increasingly important in an aging society, leading to impaired activities of daily living (ADL), frailty, and increased mortality. Although b/tsDMARDs have been reported to improve bone mass or protect against bone loss in RA, an unexpected finding from FIRST registry highlights the persistence of bone density problems in RA patients. In a 6‐month prospective observational study of 1164 patients receiving b/tsDMARDs, significant bone loss was observed despite effective disease activity suppression (mean clinical disease activity index [CDAI] reduced from 25.5 to 4.5). No specific class of b/tsDMARDs demonstrated superiority in protecting bone mass [16]. An extended study following patients for up to 5 years confirmed this phenomenon (unpublished data).

These findings suggest that while b/tsDMARDs are effective in managing RA symptoms, they do not fully mitigate the risk of osteoporosis. As a result, regular bone mineral density monitoring and proactive osteoporosis management remain essential components of RA care to ensure that bone health is prioritized alongside disease control.

## 6 Unmet Needs Highlighted by FIRST Registry

Despite significant advances in the treatment of RA, FIRST registry shows that certain unmet needs remain. First, while clinical efficacy, as measured by low disease activity (LDA) or remission, has increased over time in b/tsDMARD‐naive patients, those with prior exposure to these agents often achieve less favorable outcomes. Second, although b/tsDMARDs have improved outcomes for many RA patients, a subset continues to experience limited physical function and reduced quality of life [1].

### 6.1 Potential of JAK Inhibitors for Patients With Prior b/tsDMARD Exposure

For RA patients with prior exposure to b/tsDMARDs, FIRST registry highlights the ongoing challenge of achieving low disease activity or remission [1]. In this context, JAKi have emerged as a promising option for patients who have had an inadequate response to other therapies. A report from FIRST registry demonstrates a differential effect of bDMARDs and JAKi based on patient treatment history: while all b/tsDMARDs produced similar outcomes in b/tsDMARD‐naïve patients, JAKi showed superior outcomes compared to bDMARDs in patients with a previous b/tsDMARD failure. This advantage was even more pronounced in patients who had failed two or more b/tsDMARD classes [17, 18]. These results suggest that JAKi may offer improved disease control in difficult‐to‐treat (D2T) RA patients.

However, the safety profile of JAKi requires close monitoring and careful patient selection, particularly in elderly patients and those with comorbidities. As recommended by the European League Against Rheumatism (EULAR), a personalized approach to RA management is essential, with treatment tailored to each patient's unique clinical profile and risk factors [8]. Individualized risk assessment—including evaluation of infection risk, malignancy history, and cardiovascular health—is critical when introducing JAKi to patients with complex medical histories. By implementing these safety measures, rheumatologists can minimize adverse events and maximize the therapeutic potential of JAKi, making it a viable option for patients with prior b/tsDMARD exposure while addressing a significant unmet need in the treatment of RA.

### 6.2 Aiming for Physical Function Improvement

Another significant unmet need highlighted by FIRST registry is the limited improvement in physical function over time, even in patients with well‐controlled disease activity. Over the 20‐year history of the registry, rates of remission and LDA have increased remarkably. However, sustained improvements in HAQ‐DI scores remain elusive for certain patients. Data show that the rate of HAQ‐DI normalization after 6 months of b/tsDMARDs treatment has changed only modestly over time: from 61.4% to 63.8% in patients younger than 65 years, from 27.2% to 48.4% in patients aged 65–74 years, and from 33.3% to 43.5% in patients aged 75 years or older [1]. Considering the median HAQ‐DI in the general population of ≤ 0.06 for those aged 66–75 years, this remains an unmet need in the treatment of RA.

Strategies to bridge this gap may include: (i) early intervention, with immediate application of a T2T strategy after disease onset, and (ii) a comprehensive approach that combines non‐medical interventions such as physical training, mental health support, and nutritional counseling. Early intervention is known to lead to better outcomes. Due to ongoing discussions about the safety of JAKi in certain populations, both the FDA and EMA recommend cautious use of JAKi and recommend it primarily for Phase III: patients who have failed bDMARDs. However, JAKi generally outperforms bDMARDs in controlling disease activity and improving functional status, with faster onset of action. As persistent inflammation can lead to irreversible functional impairment, it is worth considering scenarios in which JAKi could be introduced in Phase II. Data from FIRST registry suggest that factors such as older age, high baseline HAQ‐DI, and glucocorticoid history are associated with less favorable functional outcomes in phase II/III RA patients [18]. Numerous studies have demonstrated the benefit of JAKi in improving HAQ scores, suggesting its potential value when used earlier in the treatment course for patients with these conditions.

Furthermore, FIRST registry also highlights patients who deviate from the T2T strategy by remaining on treatments that do not achieve remission—often due to safety concerns that influence both patient and clinician decisions to continue the current therapy [1]. Therefore, further identification of risk factors associated with JAKi use, as well as the development of new agents that better balance efficacy and safety, remains a crucial area of need in RA treatment.

### 6.3 Predictive Modeling for Personalized Treatment

To improve treatment outcomes, a proactive strategy using predictive modeling was explored in FIRST registry [19]. Although precision medicine in RA has been studied for a long time, it has yet to be fully implemented [20]. One of the main obstacles is the lack of reproducibility of published results, which underscores the importance of proper patient stratification before applying any precision medicine model [21]. In addition, while efficacy‐driven precision medicine models are often considered, safety‐driven precision medicine approaches are also recommended [22]. FIRST registry has prioritized safety in the selection of b/tsDMARDs, with thorough screening to identify risks. For example, CTLA4‐Ig was preferred for patients at high risk of infection, while IL‐6Ri was used for patients with a history of malignancy [1]. This approach can be considered a form of “safety‐driven precision medicine”. For patients who passed a thorough safety evaluation, we developed a precision medicine model for those without uncontrollable risk factors, allowing them to choose one of four available drug classes (TNFi, IL‐6Ri, CTLA4‐Ig, and JAKi). In FIRST registry, TNFi was the preferred choice for patients without significant risk factors who were receiving MTX [1]. The model uses machine learning techniques to predict CDAI remission in b/tsDMARD‐naive patients receiving TNFi [19]. The model considers factors such as age, baseline disease activity and functional scores to identify patients who are likely to achieve remission with TNFi therapy. Initial results are promising, showing strong predictive accuracy that allows clinicians to classify patients as potential responders or non‐responders to TNFi treatment with reasonable accuracy.

This predictive approach offers significant advantages for personalized medicine in RA. By identifying likely responders before initiating therapy, rheumatologists can optimize treatment decisions, potentially reducing the trial‐and‐error process that characterizes RA management. In addition, this model is designed to be accessible, using clinical parameters that can be easily assessed in routine practice without specialized testing [19]. This accessibility ensures that the model can be applied in different healthcare settings, facilitating its use in future RA management and in testing its reproducibility across cohorts.

### 6.4 The Importance of “In‐House Precision Medicine”

An additional benefit of the predictive modeling within FIRST registry is the concept of “in‐house precision medicine”. The predictive model was developed using clinical data readily available in daily practice, allowing institutions to build or adapt similar models based on their own patient populations. As mentioned earlier, applying a precision medicine model after proper patient stratification is one approach [21, 22], but differences in patient backgrounds between institutions can present challenges. However, if each institution develops its own model, it may enable the delivery of optimized care tailored to the specific characteristics of its patient population. This approach could serve as a reference for RA management adapted to regional or institutional characteristics. It also allows each center to refine predictive tools over time as patient data evolves, ensuring a more responsive framework for RA treatment.

By leveraging accessible technology and standard clinical parameters, “in‐house precision medicine” offers a practical method for improving treatment selection. This adaptability allows institutions to tailor RA management to the specific needs of their patients, potentially optimizing clinical outcomes while maintaining feasibility in routine practice.

## 7 Conclusion

**FIGURE 2:**
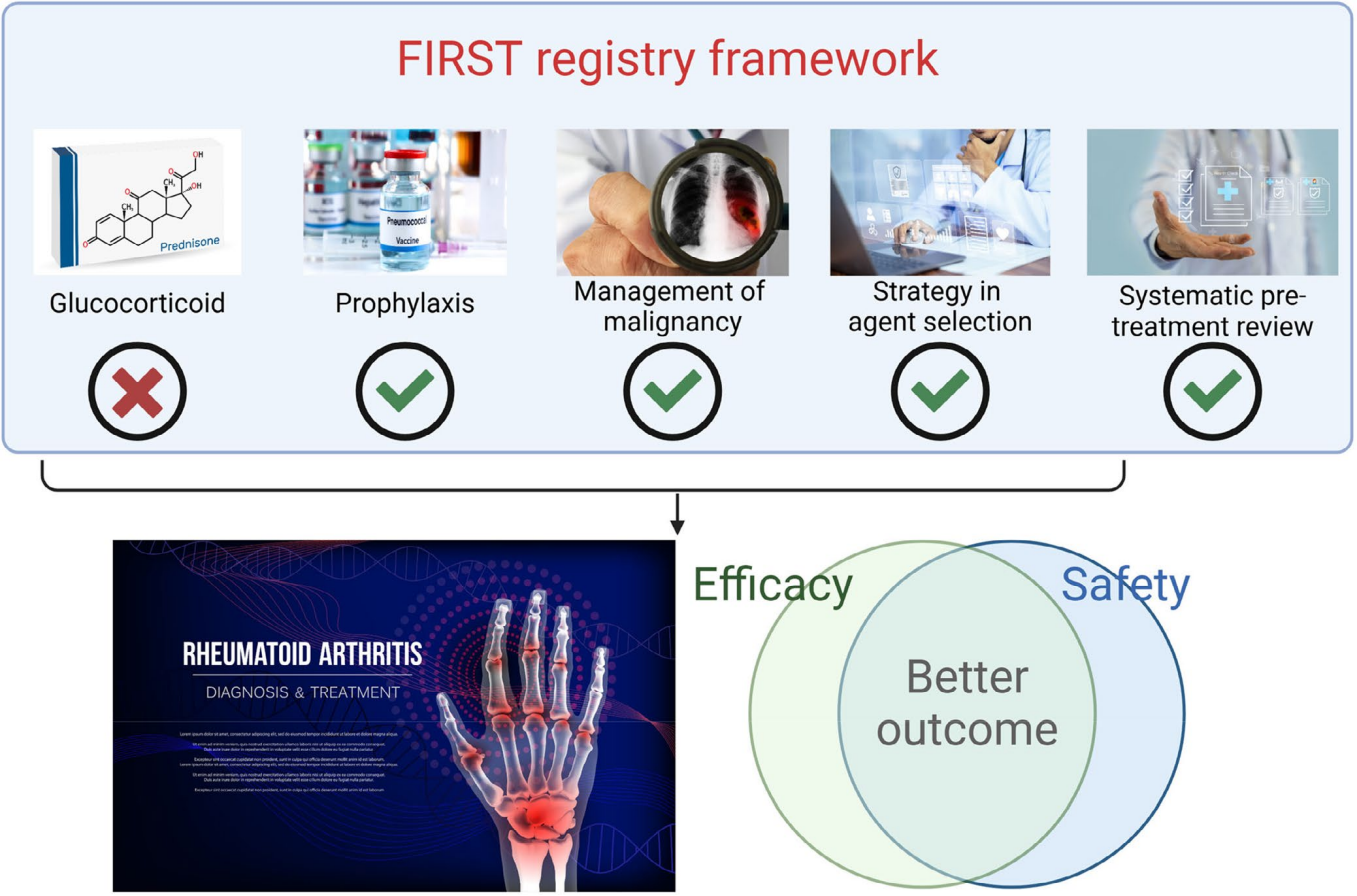
FIRST registry: 20 years of initiatives and outcomes. This figure illustrates the comprehensive approach and results of FIRST registry. The figure was created using BioRender and iStock. https://app.biorender.com/illustrations/6805d2b3c371e3ebd5a1ec64.

As RA treatment evolves in an aging population, balancing efficacy and safety is critical. FIRST registry highlights a comprehensive strategy that includes proactive infection prevention, malignancy screening, osteoporosis monitoring, and precision medicine approaches to optimize treatment outcomes (Figure 2). Risk‐based patient selection ensures timely use of b/tsDMARD while prioritizing safety for high‐risk individuals. Strategies such as PCP prophylaxis, NTM screening, vaccination and glucocorticoid reduction may have reduced serious infections, while CT screening and structured surveillance may have improved malignancy management. JAK inhibitors show promise in D2T RA, but require careful risk assessment. Predictive modeling and “in‐house precision medicine” offer a potential tool to improve treatment selection, allowing institutions to refine therapy based on their patient populations.

This review presents our approach and results in Japan, the world's oldest society. Lessons learned from this experience may help inform strategies for RA care in aging populations, contributing to efforts toward safer and more effective long‐term disease management.

## Author Contributions

Koshiro Sonomoto contributed to drafting the manuscript, Yoshiya Tanaka contributed to reviewing the manuscript. All authors read and approved the final version of the manuscript.

## Conflicts of Interest

Koshiro Sonomoto has received speaking fees from Abbvie, Eli Lilly Japan, Gilead Sciences, GlaxoSmithKline, Janssen, Pfizer Japan, UCB Japan, Astellas, Ayumi, Chugai, Taisho, Tanabe Mitsubishi, and has received research funding from UCB Japan. Yoshiya Tanaka has received speaking fees and/or honoraria from Eli Lilly, AstraZeneca, AbbVie, Gilead, Chugai, Behringer‐Ingelheim, GlaxoSmithKline, Eisai, Taisho, Bristol‐Myers, Pfizer, Taiho, and has received research grants from Mitsubishi‐Tanabe, Eisai, Chugai, Taisho.

## Data Availability

This review manuscript contains no original data.
